# Optimization of Prediction Model for Glass Transition Temperature of Thermoplastic Toughened Bismaleimide Resin

**DOI:** 10.3390/polym18091069

**Published:** 2026-04-28

**Authors:** Jindong Zhang, Yunfeng Luo, Weidong Li, Huanzhi Yang, Yichuan Zhang, Hongfei Zhou, Xiangyu Zhong, Jianwen Bao

**Affiliations:** National Key Laboratory of Advanced Composites, AVIC Composite Technology Center, AVIC Composite Corporation Ltd., Beijing 101300, China; zhangjindong@buaa.edu.cn (J.Z.); lyfarticle@163.com (Y.L.); huanzhiyang@163.com (H.Y.); zhangyc@avic.com (Y.Z.); zhouhf@avic.com (H.Z.); zxyarticle@163.com (X.Z.)

**Keywords:** bismaleimide resin, thermoplastic toughening, reaction-induced phase separation, DiBenedetto equation, model optimization

## Abstract

The brittleness of bismaleimide (BMI) resin is a major issue that restricts its use as a matrix for advanced composites. Blending with thermoplastics constitutes an effective toughening approach that preserves the thermal resistance and mechanical properties of the resin. Reaction-induced phase separation is the primary toughening mechanism in thermoplastic-toughened BMI resin. However, the complex phase-separated structure causes the relationship between the glass transition temperature (*T*_g_) and the curing degree to deviate from that described by the classical DiBenedetto equation. In this paper, two improved models, incorporating power-law correction and threshold inhibition, were constructed to address the phase-separation effect. An aerospace-grade BMI resin was toughened by a thermoplastic polyimide. The relationship between *T*_g_ and the curing degree was fitted by the DiBenedetto equation and the improved models. It was found that the adjusted coefficient of determination for the power-law correction and threshold inhibition models for the toughened resin increased to 0.978 and 0.995, respectively, whereas that of the DiBenedetto equation was only 0.612. This work provides a new, readily applicable empirical model for the prediction of *T*_g_ in thermoplastics-toughened thermosetting resins and offers theoretical support for optimizing the curing process and controlling the performance of multiphase resins.

## 1. Introduction

Bismaleimide (BMI) resin has become an important matrix material for advanced aerospace composites due to its excellent high-temperature resistance, low moisture absorption, and good mechanical properties [1,2]. Thus, it is widely used in key parts such as engine nacelles, wing skins, and high-temperature structural components [3,4]. However, unmodified BMI resin exhibits a high cross-linking density and rigid molecular chains, resulting in high brittleness and insufficient impact resistance [5,6], thereby limiting its application in high-reliability aerospace structures. Therefore, toughening modification of BMI resin is an important research topic in aerospace composites [7,8]. Among the numerous toughening methods for BMI resin [9,10,11], introducing thermoplastic polymers (such as polyetherketone, polyetherimide, and polysulfone) as a “second phase” toughener offers significant advantages. Furthermore, this method can not only significantly improve toughness, but also maintain heat resistance and mechanical properties without degradation [12,13].

For thermoplastic-toughened BMI resins, phase separation often occurs due to thermodynamic incompatibility during the curing process, a phenomenon known as reaction-induced phase separation (RIPS), thereby forming a BMI-rich phase, a thermoplastic-rich phase, and a more complex multiphase network microstructure [14]. RIPS is the most crucial toughening mechanism for thermoplastic-modified BMI resins. The thermoplastic phase can achieve toughening through mechanisms such as crack bridging, crack pinning, and crack deflection, thereby reducing stress concentrations, promoting plastic deformation, dissipating fracture energy, and impeding the propagation of microcracks [12]. For instance, Xu et al. [15] introduced polyetherketone films into the interlayers of BMI composites and induced the formation of micro-nano scaled phase-separated structures during the molding process. The compressive strength after impact was enhanced by up to 33%. In addition, processing performance and final microstructure can be optimized by regulating phase-separation behavior [15,16,17].

However, different phases caused by RIPS exhibit distinct reaction activities and segmental mobility, which produce complex effects on the evolution of glass transition temperature (*T*_g_) of the toughened resin. *T*_g_ is one of the most important thermodynamic parameters of thermosetting resins, which not only determines the upper limit of the service temperature of materials, but also directly affects their processing window, mechanical properties and long-term stability [18,19]. The quantitative relationship between *T*_g_ and the curing degree is crucial for process design and performance prediction [20,21], since *T*_g_ increases continuously as a cross-linking network forms during resin curing. However, the cross-linked network of thermoplastic-toughened BMI resin is disrupted by the thermoplastic phase, which may cause this quantitative relationship to deviate from classical theories. In addition, to improve application performance, aerospace-grade BMI resins often include other blended monomers (such as epoxy or cyanate ester) and chain extenders (such as diamine or allyl compounds), which complicates the curing reaction mechanism [9,10,11].

The classical method for predicting the quantitative relationship between *T*_g_ and the curing degree is the DiBenedetto equation [22]. DiBenedetto proposed an empirical equation that expresses *T*_g_ as a continuous function of the curing degree and has been widely used to study the glass transition in homogeneous thermosetting resins such as epoxy, cyanate ester, and benzoxazine [23,24]. However, the application of the DiBenedetto equation is based on the ideal framework of regarding the resin as a compatible system of monomers and fully cross-linked products [22,25]. Moreover, as a single-parameter model, it can only capture curvature changes in monotonic functions and is applicable to simple systems with a stoichiometric ratio of 1:1 [26,27]. Hence, for complex resins like the thermoplastic toughened BMI with excess reactants, multiple reaction mechanisms, and multiphase mixing, the DiBenedetto equation is difficult to fit the experimental data effectively.

In summary, the classical DiBenedetto equation and its existing improved forms exhibit poor predictive performance for the quantitative relationship between *T*_g_ and the curing degree in thermoplastic-toughened BMI resin. Therefore, in this paper, two new improved models were developed by embedding an “effective curing degree function” into the classical DiBenedetto equation to optimize the prediction of *T*_g_ for toughened BMI resins affected by phase separation. A self-developed thermoplastic polyimide (TPI) was used as a toughener to modify an aerospace-grade BMI resin. The evolution law of *T*_g_ with the curing degree was systematically studied using differential scanning calorimetry (DSC) results from resins cured under different thermal histories. The applicability of the classical DiBenedetto equation and the improved models to the BMI resins, both before and after toughening, was compared. The goodness of fit and the physical meaning of parameters across different models were systematically compared, combined with microstructure analysis of the scanning electron microscopy (SEM) micro-morphology of cured resins. The research aims to establish an optimized model to predict *T*_g_ of multiphase thermosetting resins and to provide a theoretical basis for optimizing the curing process of high-performance aerospace composites.

## 2. Materials and Methods

### 2.1. Raw Materials

Aerospace-grade bismaleimide resin, grade HT-280, was supplied by AVIC Composite Corporation, Ltd., Beijing, China. The TPI toughener was self-made, with basic properties detailed in the Supplementary Materials.

### 2.2. Preparation of Toughened Resin

HT-280 resin was added to a beaker and heated in a heating mantle to 60 °C with stirring. After the temperature stabilized, TPI toughener was added at 15 wt% relative to the resin and stirred at a constant temperature for 10 min to obtain the toughened modified resin, which was named HT-280G.

### 2.3. DSC Tests

**Figure 1:**
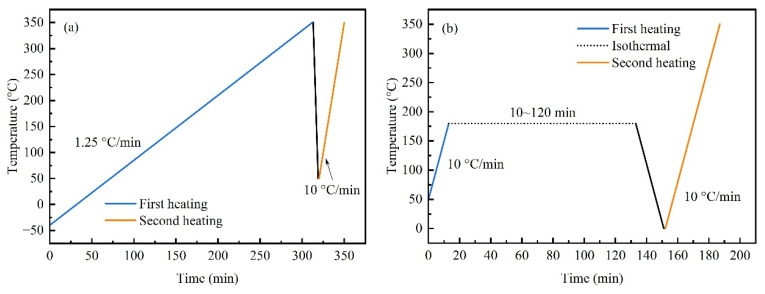
Temperature program of DSC heating scan. (**a**) Two heating scans for fresh resins; (**b**) isothermal curing and secondary heating scan.

DSC tests were performed on the resins before and after toughening using a DSC 300 differential scanning calorimeter (NETZSCH, Waldkraiburg, Germany), and the heating scan program is shown in Figure 1. Fresh resin samples weighing 5–8 mg were used in sealed aluminum crucibles under a nitrogen atmosphere at a gas flow rate of 50 mL/min.

First, two heating scans were performed on the fresh resin, and the heating scan program is shown in Figure 1a. The first scan was heated from −40 °C to 350 °C at a heating rate of 1.25 °C/min, equilibrated for 1 min, then cooled to 50 °C at a cooling rate of 50 °C/min and equilibrated for 1 min. The second scan was carried out in the temperature range of 50~350 °C at a heating rate of 10 °C/min. Among them, the *T*_g_ of the first scan was taken as the initial glass transition temperature (*T*_g0_) of the uncured resin; the integral exothermic peak area (*H*_0_) was taken as the reaction enthalpy of the fully cured resin; the *T*_g_ of the second scan was taken as the terminal glass transition temperature (*T*_g∞_) of the fully cured resin.

Then, secondary-scan results for resin cured under different thermal histories were tested, and the heating scan program is shown in Figure 1b. The fresh resin was heated to 180 °C at a heating rate of 10 °C/min and isothermally treated for 10, 20, 30, 40, 50, 60, 90, and 120 min. Then it was cooled to 0 °C at a cooling rate of 50 °C/min and equilibrated for 1 min. The secondary scan was carried out in the temperature range of 50~350 °C at a heating rate of 10 °C/min. The residual exothermic peak area (∆*H*) of the secondary scan was integrated and substituted into the following formula to calculate the curing degree of the resin cured with different thermal histories [23,27]:

(1) $$\alpha \;=\;1\;-\;∆H/H_{0},$$

### 2.4. Prediction Model of Glass Transition Temperature

The DiBenedetto equation is a classical model for studying the change of $T_{g}$ of thermosetting resins with the curing degree [20,22,23,25]:

(2) $$T_{g}(\alpha )\;={\;T}_{g0}\;+\;\lambda \alpha (T_{g\infty }-{\;T}_{g0})/(1\;-\;\alpha (1\;\;-\;\lambda )),$$

where α is the curing degree, $T_{g}(\alpha )$ is the glass transition temperature of the resin at a certain curing degree, and λ is a fitting parameter reflecting the dependence trend of $T_{g}$ on the curing degree.

However, in the thermoplastic resin-toughened BMI resin, as the curing reaction progresses, phase separation occurs due to the thermodynamic incompatibility between the two phases. The two phases may have different $T_{g}$ and different evolution modes as a function of curing degree. Therefore, the macroscopic dependence of the overall $T_{g}$ on the curing degree shows a multi-stage transition or broadened transition region, thus deviating from the prediction of the DiBenedetto equation.

Therefore, when dealing with the relationship between $T_{g}$ and the curing degree of multiphase resin systems such as thermoplastic resin toughened BMI, an attempt was made to introduce the concept of “effective curing degree ($\alpha_{\mathrm{eff}}$)” to modify the DiBenedetto equation, which corrects the phenomenon that the actual network formation efficiency is lower than the nominal curing degree caused by phase separation, local cross-linking inhomogeneity and other factors. Then Equation (2) can be rewritten as

(3) $$T_{g}(\alpha )=T_{g0}+\lambda \alpha_{\mathrm{eff}}(T_{g\infty }\;-\;T_{g0})/(1\;-\;\alpha_{\mathrm{eff}}(1\;-\;\lambda )),$$

Considering the actual physical meaning or empirical effectiveness, the following two expressions of the $\alpha_{\mathrm{eff}}$ are given to adapt to different phase separation mechanisms or fitting efficiencies:

#### 2.4.1. Power-Law Correction Model

A power function was used to correct the effective curing degree:

(4) $$\alpha_{\mathrm{eff}}\;=\;(1\;+\;r\alpha )/(1\;+\;r\alpha^{2}),$$

where r is an empirical parameter controlling the shape of the fitting curve. In general, for TPI that does not participate in the curing reaction, r < 0, making $\alpha_{\mathrm{eff}}\;<\;\alpha$ with a trend of first decreasing and then increasing. This can be explained by the fact that in the early stage of the reaction, TPI dissolved in BMI dilutes the thermosetting monomers and hinders the curing reaction. This model has a simple form and strong empirical support, making it suitable for fitting nonlinear deviations over a wide range. However, its parameters lack clear physical meaning and cannot be used to conduct a mechanistic analysis of experimental data.

#### 2.4.2. Threshold Inhibition Model

It is assumed that phase separation occurs at a certain critical curing degree ($\alpha_{c}$). Before the critical curing degree, $\alpha_{\mathrm{eff}}$ remains unchanged. After the critical curing degree is reached, the curing degree is inhibited, reducing $\alpha_{\mathrm{eff}}$. To ensure the continuity of the function, a sigmoid function was used for smoothing:

(5) $$\alpha_{\mathrm{eff}}\;=\;\alpha \;-\;(\alpha \;-\;\alpha_{c})/(1\;+\;\exp(-k(\alpha \;-\;\alpha_{c}))),$$

where k is an empirical parameter controlling the steepness of the fitting curve.

### 2.5. Preparation of Resin Casting Base

HT-280 and HT-280G resins were placed into a flat mold. Then, they were heated to 120 °C in a vacuum oven and held for 60 min at a vacuum pressure of at least 0.098 MPa. After evacuation, curing was performed following the schedule of 180 °C/2 h + 250 °C/2 h.

### 2.6. Microscopic Analysis

The impact fracture surface morphology of HT-280 and HT-280G resin casting bases was observed using a Qusttro S SEM (Thermo Fisher Scientific, Waltham, MA, USA). The accelerating voltage was 30 kV, and the sample surface was sputter-coated with gold for 90 s before the experiment.

## 3. Results and Discussion

### 3.1. Applicability of Classical DiBenedetto Equation to Untoughened and Toughened BMI Resins

**Figure 2:**
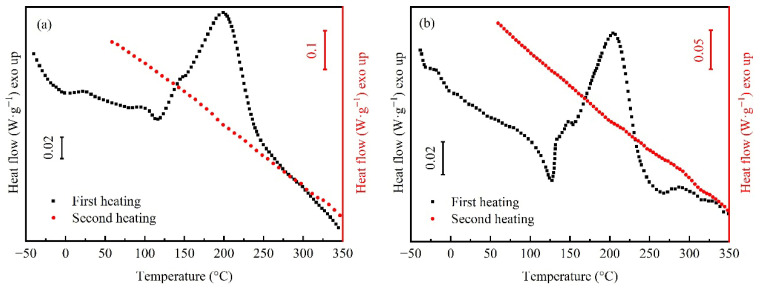
DSC heating scan curves of resins before and after toughening. (**a**) Untoughened HT-280; (**b**) Toughened HT-280G.

The two DSC heating curves of the resins before and after toughening are shown in Figure 2. From the first heating curve, the *T*_g0_ of the uncured resin and the exothermic enthalpy of the curing reaction (*H*_0_) can be obtained. After the first slow heating, the resin is considered fully cured. Therefore, the *T*_g∞_ of the cured resin can be determined from the second heating curve. The characteristic data for HT-280 and HT-280G resins were obtained from Figure 2a and b, respectively, as shown in Table 1. The uncured BMI resin is a small molecule monomer, so the *T*_g0_ of HT-280 is very low. Since TPI and BMI monomers are only in a physical blending state before curing, the high *T*_g_ of TPI will not affect the *T*_g0_ of HT-280G. Thus, it is basically consistent with that of HT-280. On the first heating curve of both resins, the endothermic peak at about 125 °C corresponds to the dissolution of BMI monomer in the allyl compound chain extender. Before this, the heat flow of HT-280G decreased continuously, which was due to the continuous dissolution of TPI in the chain extender, leading to a change in the specific heat capacity of the system. Thereafter, a main exothermic peak appears at about 200 °C, accompanied by a shoulder at about 150 °C. The low-temperature peak corresponds to the diene addition reaction between the imide ring in BMI and the allyl compound. The intermediate generated by this reaction undergoes a Diels–Alder reaction with the imide ring, corresponding to the high-temperature peak [10,11]. At a lower heating rate, the two exothermic peaks of the reaction were not completely separated. Since TPI does not participate in the curing reaction of BMI, the *H*_0_ of HT-280G is smaller than that of HT-280. Then, the *T*_g∞_ of HT-280G measured by secondary heating after full cure is slightly higher than that of HT-280, which is due to the *T*_g_ of TPI itself (see Figure S3) being slightly higher than that of HT-280 resin. Moreover, it should be noted that although only one glass transition step appears on the secondary heating curve of cured HT-280G, it is not sufficient to prove that it is a homogeneous system. As the *T*_g_ of TPI itself is close to that of HT-280, the phase structure of HT-280G needs to be further observed by micro-morphology in the following text.

**Figure 3:**
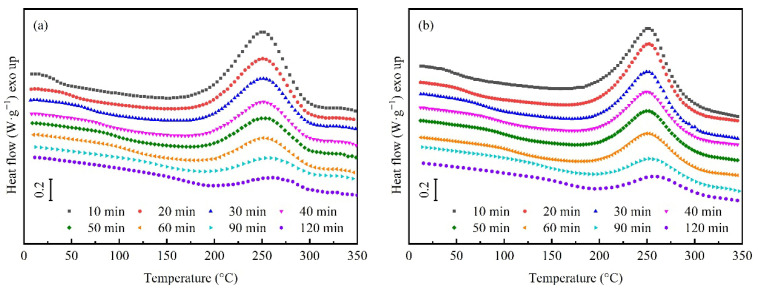
Secondary DSC scan curves of two resins cured under different thermal histories. (**a**) Untoughened HT-280; (**b**) Toughened HT-280G.

**Figure 4:**
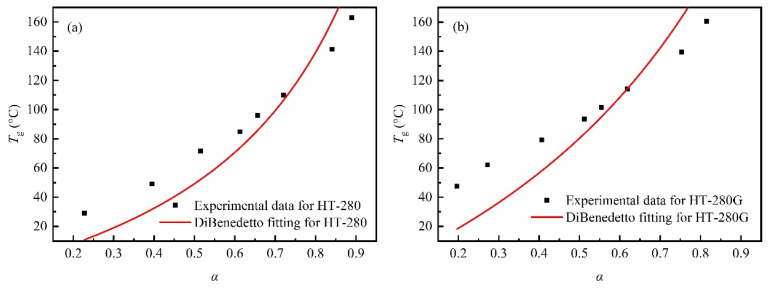
Curves of resin glass transition temperature vs. curing degree predicted by the DiBenedetto equation. (**a**) Untoughened HT-280; (**b**) toughened HT-280G.

The DSC secondary-scan curves of the resins before and after toughening, cured under different thermal histories, are shown in Figure 3. The ∆*H* was obtained by integrating the exothermic peak of the secondary scan, and the α and the corresponding *T*_g_(*α*) were calculated by substituting ∆*H* into Equation (1), as shown in Table 2. Then, the *α* and *T*_g_(*α*) data in Table 2 were substituted into Equation (2) to fit the relationship between *T*_g_ and α of the resins before and after toughening using the classical DiBenedetto equation. The results are shown in Figure 4, and the fitting undetermined coefficient λ and adjusted coefficient of determination (Adj-R^2^) are shown in Table 3. The fitting coefficients lambda for HT-280 and HT-280G are 0.23 and 0.41, respectively. In the definition of the DiBenedetto equation, λ is defined as the ratio of the segment mobility of the resin to the initial reactant at a certain reaction degree, so the value range of λ should be between 0 and 1. This value range determines the shape of the fitting curve, explaining the rapid increase in *T*_g_ with increasing cross-linking density after the gel point of the resin. However, the fit of the DiBenedetto equation to HT-280 and HT-280G is poor, with Adj-R^2^ of 0.612 and 0.827, respectively. According to the basic assumption of the DiBenedetto equation, the one-to-one correspondence between *T*_g_ and the curing degree of the resin implies that the molecular structure of the resin must remain consistent across different temperatures, which is applicable to systems in which the reactants meet the stoichiometric ratio. For aerospace-grade BMI resins such as HT-280, to obtain different cured product properties, chain extenders, accelerators that deviate from stoichiometric ratios with BMI monomers, and other process-adjustment additives may be used. Moreover, the curing reaction of the resin may involve multiple mechanisms, such as diene addition, Diels–Alder reaction, and BMI monomer self-polymerization. Therefore, its *T*_g_ may deviate from the prediction of the DiBenedetto equation. For the toughened HT-280G, the initial dissolution of TPI in the resin monomer reduces the probability of collisions among the reactive monomers. Additionally, as the curing degree increases, phase separation may reduce the cross-linking density of the BMI thermosetting phase, leading to a further deviation of *T*_g_ from the prediction of the DiBenedetto equation. Therefore, the fitting goodness of the DiBenedetto equation for HT-280G is worse than that of HT-280.

### 3.2. Applicability of Improved Models to Toughened BMI Resin

**Figure 5:**
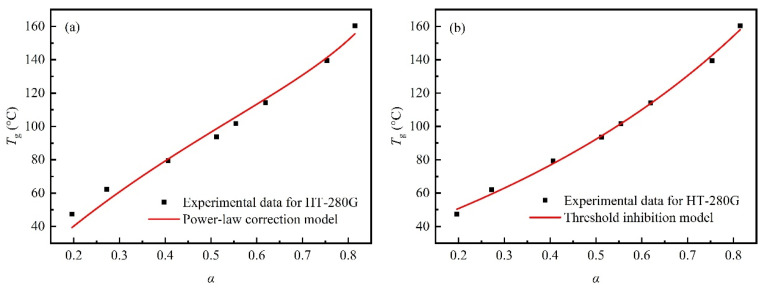
Curves of glass transition temperature vs. curing degree of toughened resin HT-280G predicted by different modified models. (**a**) Power-law correction model; (**b**) threshold inhibition model.

To accurately describe the relationship between *T*_g_ and the curing degree in heterogeneous high-toughness resins such as HT-280G, the model can be improved by adding additional empirical parameters, thereby facilitating its application to complex resin systems. The *T*_g0_(, *T*_g∞_ in Table 1 and α, *T*_g_(*α*) in Table 2 were substituted into Equation (3), and improved by the power-law correction model of Equation (4) and the threshold inhibition model of Equation (5), respectively. The improved fitting curves are shown in Figure 5, and the fitting parameters are shown in Table 4. The fitting coefficients λ for the power-law correction model and the threshold inhibition model are 0.93 and 0.26, respectively, both less than 1, consistent with their physical meaning. After adding the corresponding empirical parameters, the Adj-R^2^ are increased to 0.978 and 0.995, respectively, which are highly consistent with the experimental data. The empirical parameter r is −0.89, less than 0, resulting in the $\alpha_{\mathrm{eff}}$ being consistently lower than α. This indicates that the $\alpha_{\mathrm{eff}}$ is continuously suppressed during the curing process. In the early stage of the reaction, TPI is dissolved in the resin monomers, which reduces the collision probability of the resin monomers and thus lowers the $\alpha_{\mathrm{eff}}$. With the continuous increase in the curing degree, the TPI thermoplastic phase formed via RIPS reduces the cross-linking density of the thermoset-rich phase, also leading to a decrease in the $\alpha_{\mathrm{eff}}$. Then, the critical curing degree $\alpha_{c}$ obtained by the threshold inhibition model is 0.82. According to Equation (5), it indicates that when the curing degree exceeds this value, the $\alpha_{\mathrm{eff}}$ begins to be suppressed and deviates from the prediction of the DiBenedetto equation. This is likewise attributed to a reduction in cross-linking density due to the formation of the TPI thermoplastic phase. Compared with the power-law correction model, the threshold inhibition model provides a definite threshold for the curing degree at which phase separation occurs or exerts a significant influence on the cross-linked network. Moreover, the threshold inhibition model fits the data better than the power-law correction model. However, the power-law correction model introduces fewer empirical parameters. Hence, it has higher fitting efficiency and convenience. The *T*_g_ of the resin has a one-to-one correspondence with the curing degree, independent of the specific curing temperature. Therefore, the curing degree can be calculated using a reliable model by measuring the *T*_g_ of the cured resin to judge the rationality of the curing system, which is very important and convenient for the engineering application of resins and their composites.

**Figure 6:**
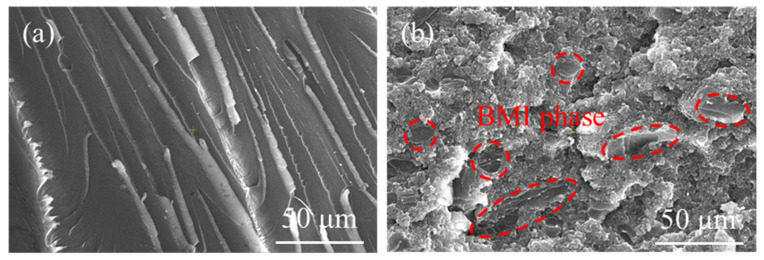
SEM images of fracture cross-section morphologies of resins before and after toughening. (**a**) Untoughened HT-280; (**b**) toughened HT-280G.

To verify that the phase separation structure of the toughened resin leads to the deviation of *T*_g_ from the DiBenedetto equation prediction, the fracture cross-section morphology of the resins before and after toughening was observed, and the SEM images are shown in Figure 6. Figure 6a shows that the cross-section of the untoughened HT-280 resin is smooth with typical brittle fracture river-like patterns, demonstrating that the resin is a homogeneous system. While in Figure 6b, the cross-section of the toughened HT-280G resin is rough with a large-area tough fracture and only a small amount of smooth cross-section (indicated by the red circles) is dispersed. The large-area tough region is the continuous TPI thermoplastic phase, while the small amount smooth region is the dispersed BMI thermosetting phase. It demonstrates that phase separation occurs during the curing of HT-280G, and that an inverted structure forms. This structure can endow the resin with high toughness. However, it is also the reason for the deviation of its *T*_g_ from the prediction of the DiBenedetto equation.

## 4. Conclusions

Aiming at the problem of insufficient fitting goodness of the classical DiBenedetto equation for non-stoichiometric reactants and multiphase resins, two improved models, including power-law correction and threshold inhibition, were established by introducing new empirical parameters to fit the relationship between *T*_g_ and curing degree of aerospace-grade BMI resin toughened by TPI. The Adj-R^2^ of the classical DiBenedetto equation for the untoughened HT-280 and the toughened HT-280G are 0.827 and 0.612, respectively. The fitting deviation of the untoughened HT-280 resin is mainly attributed to deviations in the monomer stoichiometric ratio and multiple reaction mechanisms in the BMI resin. For the toughened HT-280G resin, however, the fitting deviation is further compounded by a reduction in the cross-linking density of the thermosetting phase due to the TPI thermoplastic phase formed by RIPS. After adding the corresponding empirical parameters, the Adj-R^2^ of the power-law correction model and the threshold inhibition model for HT-280G increase to 0.978 and 0.995, respectively, which are highly consistent with the experimental data. The parameter r of the power-law correction model accounts for the reduced reaction probability of resin monomers due to dissolved TPI during the early reaction stage and the weakened cross-linking density due to the TPI thermoplastic phase during the later stage. The threshold inhibition model, on the other hand, provides a definite curing degree threshold $\alpha_{c}$ at which the TPI thermoplastic phase begins to significantly reduce the cross-linking density. The fitting goodness of the threshold inhibition model is higher than that of the power-law correction model. While the power-law correction model introduces fewer empirical parameters and offers higher fitting efficiency and convenience. In summary, two improved DiBenedetto equations have been established in this work, providing a theoretical basis for quantitatively analyzing the relationship between *T*_g_ and the curing degree of thermoplastic-toughened resins with complex compositions and phase structures, thereby facilitating the optimization of the curing process for aerospace composites.

## Figures and Tables

**Table 1:** Initial and terminal glass transition temperatures and curing reaction enthalpy of resins before and after toughening.

Sample	Initial Glass Transition Temperature, *T*_g0_ (°C)	Terminal Glass Transition Temperature, *T*_g∞_ (°C)	Curing Exothermic Enthalpy, *H*_0_ (J·g^−1^)
HT-280	−12.1	297.5	325.1
HT-280G	−10.1	303.2	290.6

**Table 2:** Residual curing enthalpy, calculated curing degree and corresponding glass transition temperature of two resins cured under different thermal histories.

Sample	Residual Exothermic Enthalpy ∆*H* (J·g^−1^)	Curing Degree, *α*	Glass Transition Temperature, *T*_g_ (*α*) (°C)
HT-280-180-10	251.0	0.228	29.1
HT-280-180-20	196.8	0.395	49.0
HT-280-180-30	157.5	0.516	71.7
HT-280-180-40	125.7	0.613	84.8
HT-280-180-50	111.8	0.656	95.8
HT-280-180-60	90.9	0.721	109.7
HT-280-180-90	51.7	0.841	141.2
HT-280-180-120	35.9	0.890	162.8
HT-280G-180-10	233.6	0.196	47.4
HT-280G-180-20	211.5	0.272	62.1
HT-280G-180-30	172.5	0.406	79.3
HT-280G-180-40	141.7	0.512	93.6
HT-280G-180-50	129.5	0.554	101.6
HT-280G-180-60	110.6	0.619	114.1
HT-280G-180-90	71.6	0754	139.4
HT-280G-180-120	53.8	0.815	160.4

**Table 3:** Fitting parameters and accuracy of resin glass transition temperature before and after toughening predicted by the DiBenedetto equation.

Sample	*λ*	**Adj-R^2^**
HT-280	0.23 ± 0.026	0.827
HT-280G	0.41 ± 0.055	0.612

**Table 4:** Fitting parameters and accuracy of the relationship between glass transition temperature and curing degree of toughened resin HT-280G predicted by different modified models.

Model	*λ*	*r*	*α*_c_	*k*	Adj-R^2^
Power-law correction	0.93 ± 0.053	−0.89 ± 0.033	—	—	0.978
Threshold inhibition	0.26 ± 0.030	—	0.82 ± 0.041	0.27 ± 0.21	0.995

## Data Availability

The original contributions presented in this study are included in the article/Supplementary Material. Further inquiries can be directed to the corresponding authors.

## Supplementary Materials

The following supporting information can be downloaded at: https://www.mdpi.com/article/10.3390/polym18091069/s1. Figure S1: The FT-IR spectrum of the self-made TPI tougher. Figure S2: The TGA curves of the self-made TPI tougher. Figure S3: The DSC curve of the self-made TPI tougher. Reference [28] is cited in the Supplementary Materials.
