# Supplementary material for: Optimization of Prediction Model for Glass Transition Temperature of Thermoplastic Toughened Bismaleimide Resin
Source: Polymers (Basel). 2026 Apr 28;18(9):1069. doi: 10.3390/polym18091069 (PMC13165353; doi:10.3390/polym18091069)
Supplement: Supplementary file 1 [file polymers-18-01069-s001.zip › polymers-4254409-supplementary.pdf]

Supplementary information: Additional data on characterization and properties

Optimization of Prediction Model for Glass Transition Temperature of Thermoplastic Toughened Bismaleimide Resin

Jindong Zhang, Yunfeng Luo, Weidong Li*, Huanzhi Yang, Yichuan Zhang, Hongfei Zhou, Xiangyu Zhong, Jianwen Bao*

National Key Laboratory of Advanced Composites, AVIC Composite Technology Center, AVIC Composite Corporation Ltd., Beijing 101300, P. R. China; zhangjindong@buaa.edu.cn (J.Z.); lyfarticle@163.com (Y.L.); huanzhiyang@163.com (H.Y.); zhangyc@avic.com (Y.Z.); zhouhf@avic.com (H.Z.); zxyarticle@163.com (X.Z.); bjwarticle@163.com (J.B.)

*Corresponding author: liwdhappy@163.com; bjwarticle@163.com

-Figure S1 The FT-IR spectrum of the self-made TPI tougher.

-Figure S2 The TGA curves of the self-made TPI tougher.

-Figure S3 The DSC curve of the self-made TPI tougher.

The chemical structure of the TPI toughener was characterized by a Tensor II Fourier transform infrared (FT-IR) spectrometer (Bruker, USA). The transmission scanning mode was adopted with a scanning range of 400~4000 cm^{-1}. The powder sample was ground and mixed with KBr, pressed into pellets under a halogen lamp, and then subjected to the test. The KBr dispersant was dried in a vacuum oven at 120 °C for 2 h before use.

The FT-IR spectrum is presented in Figure S1. The characteristic peaks of the amide bond near 1550 cm^{-1} and 1665 cm^{-1} disappeared. Meanwhile, the following characteristic peaks appeared: the symmetric stretching vibration peak of the carbonyl group at 1781 cm^{-1}, the asymmetric stretching vibration peak of the carbonyl group at 1719 cm^{-1}, the bending vibration peak of the carbonyl group at 720 cm^{-1}, and the stretching vibration peak of the amido group at 1369 cm^{-1} [28]. These results confirmed the successful synthesis of the TPI toughener.

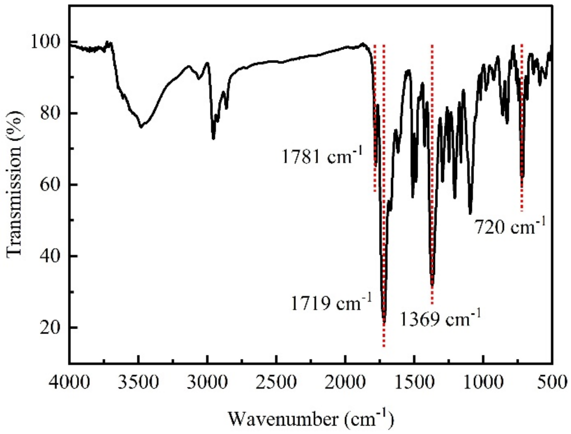

Figure S1 The FT-IR spectrum of the self-made TPI tougher.

The thermogravimetric analysis (TGA) of the TPI toughener was characterized by a TGA 550 thermogravimetric analyzer (TA, USA). The tests were conducted under air and nitrogen atmospheres, respectively, with a heating rate of 10 °C/min and a temperature range of 40~800 °C.

The TGA curves are displayed in Figure S2. The 5% thermal decomposition temperatures of the TPI toughener under air and nitrogen atmospheres were 456 °C and 502 °C, respectively, indicating its excellent heat resistance. It is thus verified that this TPI is suitable for use as a toughener for aerospace-grade bismaleimide resins, meeting their high-temperature resistance requirements in service.

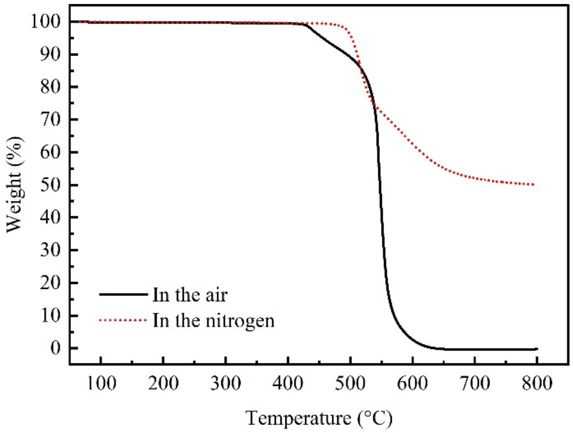

Figure S2 The TGA curves of the self-made TPI tougher.

The differential scanning calorimetry (DSC) test was performed using a DSC 300 differential scanning calorimeter (NETZSCH, Germany). TPI powders weighing 5-8 mg were used in sealed aluminum crucibles under a nitrogen atmosphere at a gas flow rate of 50 ml/min. Two heating scans were performed on the sample. The first scan was heated from 40 °C to 350 °C at a heating rate of 10 °C/min and equilibrated for 1 min to erase its thermal history. Then, the sample was cooled to 40 °C at a cooling rate of 50 °C/min and equilibrated for 1 min. The second scan was carried out in the temperature range of 40~350 °C at a heating rate of 10 °C/min.

The DSC curve is shown in Figure S3. The T_g of TPI itself is about 315 °C, which is slightly higher than that of HT-280 resin. Therefore, toughening HT-280 resin with TPI will not reduce the T_g of the resin. Meanwhile, the T_g of the resin will not exhibit differentiation due to environmental factors such as heat and humidity. In addition, a small endothermic peak can be observed superimposed on the glass transition step, attributable to the melting of a small amount of TPI crystals.

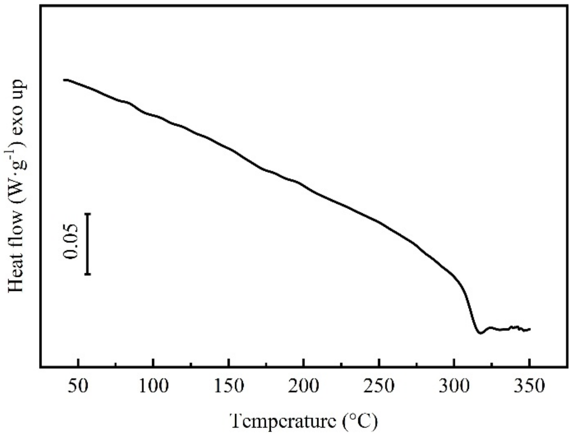

Figure S3 The DSC curve of the self-made TPI tougher.
